# Bile Microbiome Signatures Associated with Pancreatic Ductal Adenocarcinoma Compared to Benign Disease: A UK Pilot Study

**DOI:** 10.3390/ijms242316888

**Published:** 2023-11-28

**Authors:** Nabeel Merali, Tarak Chouari, Julien Terroire, Maria-Danae Jessel, Daniel S. K. Liu, James-Halle Smith, Tyler Wooldridge, Tony Dhillon, José I. Jiménez, Jonathan Krell, Keith J. Roberts, Timothy A. Rockall, Eirini Velliou, Shivan Sivakumar, Elisa Giovannetti, Ayse Demirkan, Nicola E. Annels, Adam E. Frampton

**Affiliations:** 1 Minimal Access Therapy Training Unit (MATTU), Royal Surrey County Hospital NHS Foundation Trust, Egerton Road, Guildford GU2 7XX, UK; 2 Department of Hepato-Pancreato-Biliary (HPB) Surgery, Royal Surrey County Hospital NHS Foundation Trust, Egerton Road, Guildford GU2 7XX, UK; 3 Section of Oncology, Department of Clinical and Experimental Medicine, Faculty of Health and Medical Science, University of Surrey, Guildford GU2 7WG, UK; 4 Surrey Institute for People-Centred AI, University of Surrey, Guildford GU2 7XH, UK; 5 Section of Statistical Multi-Omics, Department of Clinical and Experimental Medicine, Faculty of Health and Medical Science, University of Surrey, Guildford GU2 7WG, UK; 6 Division of Cancer, Department of Surgery and Cancer, Imperial College London, Hammersmith Hospital Campus, London W12 0NN, UK; 7 Hepatobiliary and Pancreatic Surgery Unit, Queen Elizabeth Hospital Birmingham, College of Medical and Dental Sciences, University of Birmingham, Birmingham B15 2TH, UK; 8 Department of Life Sciences, Imperial College London, London SW7 2AZ, UK; 9 Centre for 3D Models of Health and Disease, Division of Surgery and Interventional Science, University College London (UCL), London W1W 7TY, UK; 10 Oncology Department, Institute of Immunology and Immunotherapy, Birmingham Medical School, University of Birmingham, Birmingham B15 2TT, UK; 11 Department of Medical Oncology, VU University Medical Center, Cancer Center Amsterdam, 1081 HV Amsterdam, The Netherlands; 12 Cancer Pharmacology Lab, AIRC Start Up Unit, Fondazione Pisana per la Scienza, San Giuliano Terme PI, 56017 Pisa, Italy

**Keywords:** pancreatic cancer, microbiome, 16S rRNA gene, bile, biomarker

## Abstract

Pancreatic ductal adenocarcinoma (PDAC) has a very poor survival. The intra-tumoural microbiome can influence pancreatic tumourigenesis and chemoresistance and, therefore, patient survival. The role played by bile microbiota in PDAC is unknown. We aimed to define bile microbiome signatures that can effectively distinguish malignant from benign tumours in patients presenting with obstructive jaundice caused by benign and malignant pancreaticobiliary disease. Prospective bile samples were obtained from 31 patients who underwent either Endoscopic Retrograde Cholangiopancreatography (ERCP) or Percutaneous Transhepatic Cholangiogram (PTC). Variable regions (V3–V4) of the 16S rRNA genes of microorganisms present in the samples were amplified by Polymerase Chain Reaction (PCR) and sequenced. The cohort consisted of 12 PDAC, 10 choledocholithiasis, seven gallstone pancreatitis and two primary sclerosing cholangitis patients. Using the 16S rRNA method, we identified a total of 135 genera from 29 individuals (12 PDAC and 17 benign). The bile microbial beta diversity significantly differed between patients with PDAC vs. benign disease (Permanova *p* = 0.0173). The separation of PDAC from benign samples is clearly seen through unsupervised clustering of Aitchison distance. We found three genera to be of significantly lower abundance among PDAC samples vs. benign, adjusting for false discovery rate (FDR). These were *Escherichia* (FDR = 0.002) and two unclassified genera, one from *Proteobacteria* (FDR = 0.002) and one from *Enterobacteriaceae* (FDR = 0.011). In the same samples, the genus *Streptococcus* (FDR = 0.033) was found to be of increased abundance in the PDAC group. We show that patients with obstructive jaundice caused by PDAC have an altered microbiome composition in the bile compared to those with benign disease. These bile-based microbes could be developed into potential diagnostic and prognostic biomarkers for PDAC and warrant further investigation.

## 1. Introduction

Pancreatic cancer (pancreatic ductal adenocarcinoma, PDAC) is a devastating disease. It is projected that by 2030, PDAC will become the 2nd leading cause of cancer-related death [1]. The incidence and mortality rates of PDAC are increasing. Poor outcomes are partly due to late diagnosis and these patients have either inoperable local disease or incurable metastatic disease. As a result, most patients are ineligible for surgery, and systemic treatments are not sufficient. Even after potentially curative surgical resection, the recurrence rates are very high. Optimal surgery and adjuvant chemotherapy results in a median disease-free survival (mDFS) of 13.9 months (range 12.1–16.6) with gemcitabine and capecitabine [2]; and 21.6 months (range 17.7–27.6) with FOLFIRINOX (FOLFIRINOX = Folic acid, Fluorouracil, Irinotecan and Oxaliplatin) [3]. Indeed, despite advances in surgical and oncological treatments, 5-year overall-survival (OS) is only 6% [4].

The tumour microbiome is gaining more interest recently in terms of prognosis and response to therapy. We now know the pancreas is not necessarily a sterile organ and can be infected by the gut microbiome refluxing into the pancreatic duct by the upper gastrointestinal tract [5,6]. Studies have shown that the pancreatic intra-tumoural microbiome can influence tumourigenesis, chemoresistance and the patients’ immune response to the cancer [5,7,8,9,10,11,12]. Furthermore, specific microbes, such as *Gammaproteobacteria*, can inactivate gemcitabine chemotherapy leading to worse survival in PDAC mouse models [8]. Riquelme et al. disclosed the intra-tumoral microbiome composition of PDAC patients and identified a specific intra-tumoral microbiome signature (*Pseudoxanthomonas-Streptomyces-Saccharopolyspora-Bacillus clausii*) predicting the long-term survivorship of PDAC [9].

Assessing the influence of the microbiota in human physiology has revolutionised our understanding of medicine. Nejman et al. found that intra-tumoral microbiome composition is diverse and cancer type-specific [13]. The presence of bacteria in the pancreas can stimulate resident leukocytes to produce Interleukin-1β (IL-1β)*,* which produces proangiogenic factors in the tumour microenvironment (TME) (e.g., Vascular Endothelial Growth Factor (VEGF), Tissue Necrosis Factor (TNF)) [14]. Das et al. demonstrate that tumour-derived IL-1β is required for the establishment of the immunosuppressive pancreatic TME [15]. This weakens the host immune defence system by the activation of inflammatory pathway mediators; Toll-like receptors (TLRs) and microorganism-associated molecular patterns (MAMPs) that leads to bacterial trans-location and chronic inflammation. IL-6 is an important proinflammatory cytokine that leads to tumour progression. A recent study identified *Helicobacter pylori* (*H. pylori*), can alter the expression of IL-6 by microRNA regulation [16] and can induce contact between leukocytes and other microorganisms [17]. Pushalkar et al. showed the depletion of the gut microbiome led to a reduction of Myeloid-derived suppressor cells (MDSC) infiltration and reprogramming of Tumour-associated macrophages (TAMs) toward a tumour-protective M1-like phenotype. Therefore, ablation of the gut microbiome highlighted T Helper 1 Cells 1 (Th1) polarization of Cluster of Differentiation 4 (CD4+) T cells and enhanced the cytotoxic phenotype of CD8+ T cells [5]. Certain microbes can cause genotoxic effects (i.e., colibactin) that damage the host DNA and activates IL-23-producing myeloid cells that promote tumour growth [18]. The microbiome can also modulate innate and adaptive immune responses to further contribute to the formation of the immunosuppressive TME found in PDAC [19].

The bile is potentially a rich source of novel biomarkers for PDAC and Biliary tract cancers (BTC) due to its intimate proximity to the malignant lesion. The bile duct was once considered a sterile environment. However, it is now well-regarded that microbiota exists within the bile duct in both benign and malignant diseases of the hepato-pancreato-biliary system, as well as other diseases of the alimentary canal [20,21,22,23,24,25,26,27]. Over the last two decades, several studies have explored the bile microbiota in the context of benign biliary tract disease [21,28,29]. Yet there is a paucity of studies investigating the bile microbiome in the context of PDAC [22,23,26,30].

A recent study has shown that the bile does have a distinct microbial fingerprint in PDAC, as compared to other pancreatic biliary diseases [31]. Furthermore, alteration of the bile microbiome can have a direct effect on the pancreatic cell survival [28]. Therefore, investigating the intra-tumoral microbiome through the role played by the bile microbiota in biliary cancers is the next frontier in clinical cancer treatment. Given the high fatality rate and the silent progression of early disease, identifying risk factors for the prevention and early detection of biliary tract cancers is critical. Therefore, the aim of this work was to define differentiating bile microbial signatures in patients presenting with obstructive jaundice caused by PDAC and benign pancreaticobiliary disease.

## 2. Results

### Patient Characteristics

A total of 31 patients were enrolled in the study, corresponding to PDAC (*n* = 12) and benign cases (*n* = 19). Unfortunately, two benign samples were excluded due to low read counts for analysis as we did not have enough bile volume and insufficient DNA quality. Therefore, reliable data was available and analysed from only 12 PDAC and 17 benign cases (10 cases of common bile duct stones, six cases of gallstone pancreatitis, and one patient with primary sclerosing cholangitis). Table S1 shows a summary of the patient cohort.

All the PDAC cases had tumours in the head of the pancreas and were stented. The common aetiology found at Endoscopic Retrograde Cholangiopancreatography (ERCP) in the benign cases were for Common Bile Duct (CBD) stones and benign inflammatory strictures. There were an equal number of cholangitic patients in each group with similar median C-Reactive Protein (CRP) values at the time of ERCP, with no significance between the groups. The PDAC group presented with worsening jaundice, identified with statistically significant bilirubin and Carbohydrate Antigen 19-9 (CA19-9) tumour markers. Only two benign cases had a course of antibiotics within the previous month for the management of a bile leak.

**Figure 1:**
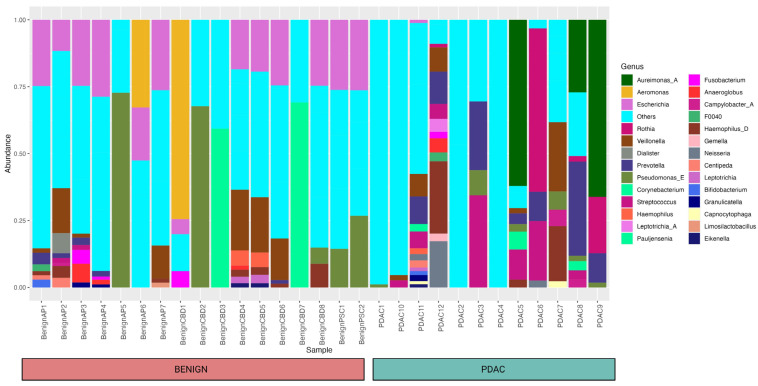
Bar plot showing the relative abundance of different bacteria within each sample and cohort at the genus level. A total of 135 genera from 29 individuals (12 PDAC and 17 benign) were identified, and a relative abundance of 10% and above was included in the figure. Forty-one different taxa that were not mapped to any bacteria at the genus level were clustered under “others” for the simplicity of this figure.

Using 16S rRNA gene analysis, we identified a total of 135 genera from 29 individuals (12 PDAC and 17 benign) and their relative abundances are shown in Figure 1.

We only used the taxa identified at the genera level for our research. As seen in the bar plot, this occurred for a few samples that had all taxa identified as “known” at the genus level. Whereas 41 different taxa were not mapped to any bacteria at the genus level and clustered under “others”. These taxa were included as separate entities in the differential abundance analysis.

**Figure 2:**
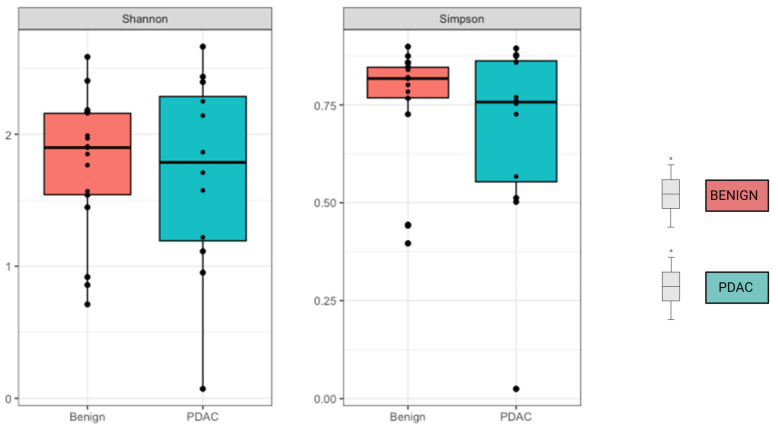
No difference was observed in Alpha diversity using the Shannon and Simpson index. Mean alpha diversity is higher among the benign samples, with *p*-values of 0.31 for Shannon and 0.3 for Simpson indices.

**Figure 3:**
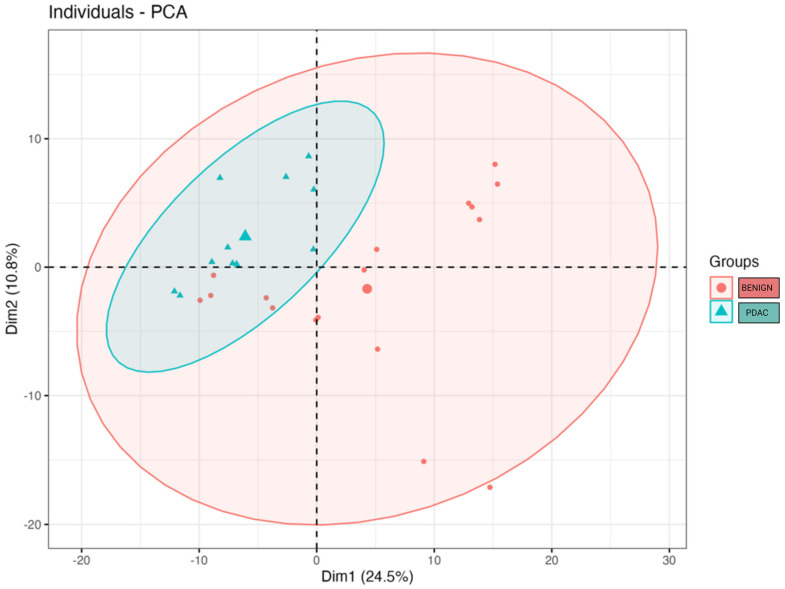
Microbial beta diversity significantly differs between bile from individuals with PDAC vs. bile from individuals with benign samples (*p* = 0.0173). The clusters were visualised by plotting the first two components that explain up to 35.3% of the variation in the sample space. The large blue triangle and the large red dot represent the centroids of the PDAC and benign sample groups.

We compared the alpha and beta diversity of the bacterial communities per group (PDAC vs. Benign). Alpha diversity did not significantly differ between sample groups (Figure 2). In regard to beta diversity, we used Aitchison distances as the measure of inter-sample differences in the compositions of gut metagenomes, which revealed a significant difference in average microbiome composition between bile from individuals with PDAC compared to individuals with benign samples by PERMANOVA (*p* = 0.0173) (Figure 3).

**Figure 4:**
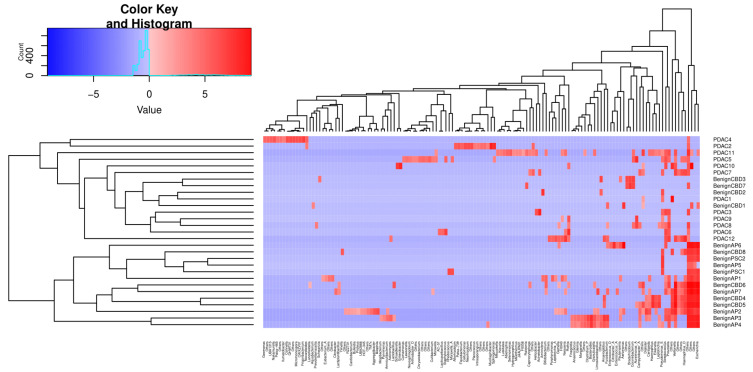
Unsupervised clustering of selected genera abundances separates PDAC from benign samples. The phylogenic tree clustering learned from 135 genera is shown in the *x*-axis. The *y*-axis shows the individual samples, which are separated into two major clusters, as shown by the dendrogram on the left. The colour key code is a graphic representation of centre-log ratio (CLR) abundance that uses all taxon read counts within a sample divided by this geometric mean, and the log fold changes in this ratio between samples are compared. If the abundance of a bacteria is lower than the mean CLR value, then it will be negative (blue), and if it is higher, then it will be positive (red).

We then performed unsupervised clustering of the PDAC and benign groups’ metagenomes based on Canberra distances of CLR-transformed abundance counts, as shown in Figure 4. The first cluster identified consists of 16 samples, 12 of which are PDAC, whereas no PDAC samples were assigned to the second cluster of 13 samples.

We next tested the differences in the relative abundance of microbial communities between PDAC and benign samples, using Maaslin2 default parameters. We found three genera to be of significantly lower abundance among PDAC samples compared to benign after adjusting for false discovery rate (FDR). These were *Escherichia* (FDR = 0.002), an unclassified genus from *Proteobacteria* (FDR = 0.002) and an unclassified genus from *Enterobacteriaceae* (FDR = 0.011). In the same samples, the genus *Streptococcus* (FDR = 0.033) had increased abundance in the PDAC group. This has been summarised in Table S2. Our data is compatible with Minimum information about a marker gene sequence (MIMARKS) and minimum information about any (x) sequence (MIxS) specifications [32] and is summarised as a checklist in Table S3.

## 3. Discussion

PDAC is an aggressive cancer with a high risk of invasion and metastasis. Furthermore, they are resistant to most cytotoxic agents and are often diagnosed at advanced stages. In the absence of an obvious mass lesion on cross-sectional imaging, determining the benign or malignant nature of a biliary stricture is important and can be even more challenging [33]. Evaluation of indeterminate strictures typically involves cytological and histological assessment. Biliary brush cytology and intraductal biopsies that are routinely performed during ERCP to assess malignant-appearing biliary strictures are limited by relatively low sensitivity [34]. It is critical to establish new diagnostic, prognostic, and therapeutic biomarkers which can complement the cytological and histological assessment of such strictures as well as any therapeutic strategies. One potential avenue of study is bile biomarkers. Bile is a potentially rich source of novel biomarkers for PDAC due to its intimate proximity to pancreatic parenchyma, which can be readily acquired via ERCP. This may prove valuable in the assessment of the underlying aetiology of biliary strictures. Emerging studies have revealed the role of the microbiome as a causative, prognostic, and predictive factor in various cancers and their treatment, including but not limited to PDAC [35]. Therefore, investigating the bile microbiota in PDAC may be the next frontier in diagnostics, prognostication, and management strategies.

Using a targeted amplicon sequencing approach for 16S rRNA gene to investigate the bile microbiome in PDAC, we have demonstrated that patients with obstructive jaundice secondary to PDAC, have an altered microbiome in the bile, compared to those with benign disease. We have identified four statistically significant microbes that are associated with PDAC. This study confirms the growing body of evidence that high microbial diversity is present within the biliary milieu of patients with benign and malignant pancreaticobiliary conditions [22,23,24,26,36,37,38].

For example, previous studies have evaluated the oral, gut, bile and/or intra-tumoural microbiota in relation to PDAC, and have found links between *Escherichia*, *Proteobacteria, Enterobacteriaceae* and *Streptococcus* [22,25,30,39,40]. Nagata et al. found an enrichment of *Streptococcus* spp. in the gut microbiome of PDAC patients [39]. A further study by Chen et al. also found *Streptococcus* as one of the gut pathogenic genera that exhibited a significant increase in abundance in patients with pancreatic cancer [25]. Previous in vitro and in vivo work has showcased *Streptococcus* can modify the biological effects of bile on PDAC cancer cell survival [28]. Our results also suggest an increased relative abundance of the genus *Streptococcus* in the bile of PDAC patients. The work described above may support the hypothesis that retrograde translocation of certain gut microbiome constituents into the CBD may have implications on cancer cell survival in PDAC. A larger study correlating the relationship between gut and bile microbiome analysis and clinical outcomes should be considered.

It should be noted that other work has drawn some conflicting results. For example, Li et al. recently investigated the microbiome differences among 53 patients with benign and malignant hepato-pancreato-biliary tract diseases. They found specific microbial bile markers for various malignant and benign disease states. *Streptococcus* was actually identified as a marker for distal cholangiocarcinoma (dCCA) and not PDAC. In vitro work has also shown *Streptococcus* has a pathognomic role in disease progression in Primary Sclerosing cholangitis to biliary dysplasia [27]. Interestingly, in PDAC, they found 24 microbial biomarkers at a genus level, none of which are in keeping with the 4 markers found in our study. The 3 most abundant markers for pancreatic cancer included *Pseudomonas, Chloroplast* and *Acinetobacter*, compared to other etiologies [26].

Our work demonstrates a low relative abundance of Escherichia and Enterobacteriaceae at a genus level. In a similar vein, work has shown that patients diagnosed with PDAC were associated with more bactibilia and *Escherichia* spp. was a negative predictor of PDAC [40]. Yet other work has found somewhat contradictory findings. For example, *Escherichia coli* [41] and *Escherichia-Shigella* [41] were found to be abundant in the biliary microbes of PDAC patients versus their benign counterparts [28]. Maekawa et al. investigated the presence of bacteria in pancreatic juice samples (taken post-operatively from drainage tubes) and found that *Enterobacter* and *Enterococcus* spp. were the major microbes in patients with PDAC [30]. Poudel et al. explored ERCP-derived bile microbial signatures in 46 patients with either PDAC, Cholangiocarcinoma (CCA), gallbladder cancer or benign biliary tract pathology. They demonstrated a distinct bile microbiome signature capable of differentiating all malignant pancreatic-biliary disease from benign disease samples. In fact, they identified a predominance of genera *Dickeya*, *Eubacterium hallii group*, *Bacteroides*, *Faecalibacterium*, *Escherichia-Shigella* and *Ruminococcus 1*, in bile samples from pancreato-biliary malignancies as compared to benign disease [22]. This study also highlighted a distinct dysbiosis not only between pancreaticobiliary cancers and benign disease but between different malignancies of the pancreaticobiliary system. Unfortunately, they have not compared subgroup bile microbiome profiling of PDAC versus benign disease. The studies described above have drawn some similar conclusions to our work yet other contradictory findings.

The conflicting findings of such studies [26] may be due to several reasons. We must consider that there are nuances to the bile microbiome we do not yet understand relating to environmental, host and tumour factors. Studies have previously shown certain clinical variables may be associated with significant changes in specific microbiota abundance found in bile, whilst other factors are of no significance [24]. Unfortunately, there is heterogeneity in terms of available clinical information and exclusion criteria in the studies described above. For example, Li et al. excluded patients with other systemic diseases, previous neoplastic disease or those receiving proton-pump inhibitors/antibiotics/prebiotics within one month [26]. Kirishima et al. did not specify if patients received antimicrobials whilst some patients had received chemotherapy [23]. These factors logically have implications on the microbiome and results observed. Patients in our study were treatment naïve. The fact patients received anti-neoplastic therapies in some of the above studies suggests their cohorts were at different stages in the patient journey once the diagnosis had already been confirmed [42]. Thus, our study may be more applicable to the initial diagnostic role of bile microbiome analysis in jaundiced patients. Furthermore, it is not entirely clear how the stage of disease or use of systemic therapies implicates the bile microbiome. Thus, making it challenging to draw any robust comparisons between our study and others. However, it is likely that heterogeneity in cohorts can explain the conflicting findings. This extends to our study as well, we have included patients with Stage I-IIA and III disease (Supplementary Materials, Table S1).

It should also be noted that previous work investigating ERCP-derived bile samples in PDAC has not clarified if patients were undergoing their first ERCP or had prior instrumentation of the CBD [22,26]. Such information is of particular importance when we consider the growing body of evidence suggesting the CBD and PDAC TME become colonized through a retrograde fashion from the duodenum [5,12,43] or after prior instrumentation. If indeed patients underwent a prior ERCP, it may in part explain some contradictory findings noted between our work and others. Furthermore, other work has used bile samples from surgically removed gallbladders [23]. It is not clear if the gallbladder-specific microbiome compartment correlates with the CBD-specific compartment. Other work has used pancreatic juice fluid sampled from surgical drains in the post operative period [30]. Again, such factors in part explain discrepancies noted between studies and between our study and others.

Nonetheless, all studies begin to fill the knowledge gap associated with the PDAC-associated bile microbiome and add value as a resource for future studies to build on. Our study has demonstrated a significant inter-sample difference in the average microbiome composition of bile in PDAC versus benign disease. However, fundamental questions remain on how we can generalise the findings of our study and contextualise it with other studies in this field. It is clear a degree of standardisation in terms of both study design and available demographic information on host, tumour and environmental factors is required in future studies to contextualise any findings observed. Emphasising the need for a further larger, comprehensive study into the four significant bacteria that we have identified in relation to PDAC as well as others identified in other studies.

Other work has alluded to the role of bile microbiome analysis in prognostication. For example, in PDAC or CCA, the relative abundance of certain microbiota correlated with prognosis after adjusting for clinicopathological variables [23]. Their results showed no common microbe correlated with a poor prognosis between tumour types. This may suggest different microbiome shifts at play within the disease-specific microenvironment, with implications on prognosis. This may have implications for clinical decision-making in the future if validated in larger studies. Follow-up with the collection of relevant clinicopathological variables in our study cohort may provide valuable insights into the correlation between bile microbiome and outcomes.

Whilst other work has demonstrated systemic therapies can alter the biliary microbiome with subsequent clinical implications. For example, S. Nadeem et al. assessed the impact of neoadjuvant chemotherapy (NAC) on the biliary microbiome in 168 patients with PDAC. Concluding that patients who received NAC exhibited significantly increased growth of Gram-negative anaerobic bacteria (*p* = 0.043), stating that perioperative antibiotic prophylaxis should be tailored to cover Gram-negative organisms and *enterococci* [42]. A direct pathological role for the bile microbiome has yet to be established. However, a previous study attributed the reduced abundance of oral *Streptococcus mitis* in PDAC to the protective role it plays against cariogenic bacterial adhesion [44]. This may result in a loss of colonisation by *Streptococcus* spp., which is thought to contribute to aggressive periodontitis [45], a risk factor for PDAC. Other work has proposed a bacterial-induced carcinogenesis model for the PDAC [46]. Whilst pre-clinical work suggests an alteration in the bile microbiome from biliary stenting has direct implications on pancreatic cell survival [28]. Further work is required to understand the effects other microbes in the bile (or PDAC TME) may have on these 4 genera (and vice versa) as this may help create a comprehensive picture of how the microbiome impacts PDAC carcinogenesis. Metagenomic assessment of the bile microbiome may shed further light on our functional understanding of the bile microbiome in PDAC carcinogenesis.

Precautions were taken to avoid intestinal milieu contamination during ERCP collection, we cannot rule that bacteria originating from the duodenum were included in the bile, since separated milieus were not screened. Likewise, direct contamination from the endoscope route leading to the introduction of bacteria from patients’ oral and oesophago-gastric route cannot be ruled out. Furthermore, we only obtained one bile sample per patient, additional sampling in future study may further minimise the impact of contamination on findings.

Secondly, this is a single-centre research study with a small sample size, which should be expanded in the future. First, because of the non-randomized nature of the study, our study provides room for the traditional confounders of selection bias. In our study, bile was sampled at a diagnostic stage, where bile signatures correlating with diagnosis may be reflective of their potential future clinical role in diagnostics whilst also providing insights into bile microbial changes and carcinogenesis, prior to any systemic therapy or disease progression which may alter microbiome compositions. Of course, an understanding of the linear changes of the bile microbiome with duration of disease, antimicrobial/antineoplastic therapies received as well as the stage/extent of the disease is required to further contextualise this. Unfortunately, we remain at a primitive stage in our understanding of the bile microbiome in both pancreatic and biliary tract malignancy with a scarcity of studies exploring this topic. Unfortunately, a rate-limiting step in our understanding of the above, is the knowledge gap in understanding what a healthy bile microbiome entail. This is ethically challenging to ascertain and will likely prove to be a major hurdle in our understanding of the bile microbiome moving forward. Molinero et al. have tried to circumvent this hurdle by evaluating bile from liver donors without a history of biliary or hepatic disorders. They found an abundance of sequences belonging to the family *Propionibacteriaceae* in healthy controls compared to patients with cholelithiasis who had an abundance of sequences belonging to the families *Bacteroids, Prevotellaceae* and *Veillonellaceae* [21].

## 4. Materials and Methods

### 4.1. Patient Enrolment

**Figure 5:**
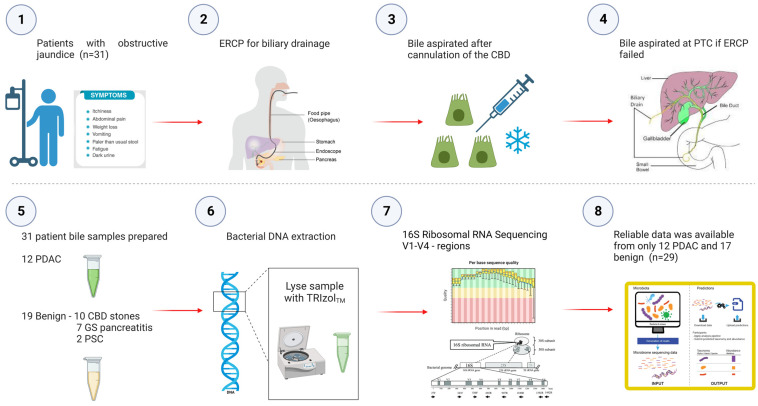
Study Flowchart outlining aspiration of bile at the time of obstructive jaundice at ERCP and subsequent sequencing of the bile microbiome. Prospective bile samples were obtained from 31 patients who underwent either ERCP or PTC. The cohort consisted of 12 PDAC, 10 choledocholithiasis, seven gallstone pancreatitis and two primary sclerosing cholangitis patients. Using the 16S rRNA method, we identified a total of 135 genera from 29 individuals (12 PDAC and 17 benign).

A prospective, non-randomised study in which 31 patients undergoing their first endoscopic retrograde cholangio-pancreatography (ERCP) at the time of obstructive jaundice for benign and malignant pancreatico-biliary disease/strictures were recruited and assessed for their microbial signatures in biliary fluid (Figure 5).

Patient biospecimens and clinical information were obtained from participants enrolled in microRNAs as BILE-based biomarkers in the Pancreaticobiliary Cancers (MIRABILE) research project. The pathological diagnoses were performed by NHS pathologists. Clear notice and signed written informed consent was obtained from all participants of this research (ICHTB HTA ethics license 12275/REC Wales approval: 17/WA/0161).

The patients were included according to the following inclusion criteria: (1) Age ≥ 18 years; (2) WHO performance status 0, 1 or 2; (3) willing and mentally able to provide written informed consent; (4) presented with obstructive jaundice and an indeterminate biliary stricture; (5) known benign or malignant pancreaticobiliary disease and undergoing their first ERCP or percutaneous transhepatic biliary drainage (PTBD). The exclusion criteria were as follows: (1) No clinical or image data suggestive of pancreaticobiliary disease and no need for endoscopic intervention or investigation; (2) pregnant women; (3) patients undergoing ERCP post-bariatric surgery, hepatico-jejunostomy or Bilroth II surgery; (4) any infectious disease such as hepatitis or HIV (human immunodeficiency virus). Side-viewing endoscopes, which were strictly sterile before the operation, were used to keep their working channel sterile. New plugs for the working channel were applied to every patient. To avoid contact and contamination with the duodenal mucosa upon ERCP, once the endoscope canal exit was positioned to the biliary duct entry, a biliary catheter was used for bile duct canalization and 5 mL of bile was aspirated in the lower third of CBD through the sphincterotome and into a sterile syringe. We obtained the bile before contrast injection, brushing and stenting in all subjects enrolled in our study. If the ERCP procedure was abandoned or technically not possible and the patient underwent PTBD, then bile will be aspirated from the drain bag after the procedure. After aspiration, bile was then frozen in our tissue bank at −80 °C until DNA extraction.

### 4.2. DNA Extraction, Amplification, and Sequencing

The Invitrogen™ TRIzol^®^ LS Reagent, method [47] was employed for the extraction of total genomic DNA. The bile was vortexed and centrifuged at 300× *g* for 10 min at 4 °C to remove cells and debris. The supernatant was then transferred to a sterile tube and centrifuged at 16,000× *g* for 10 min at 4 °C to remove further debris. An amount of 1 mL of filtered bile was transferred into a sterile 5 mL container, and 3 mL Invitrogen™ TRIzol^®^ LS reagent was added (3:1), followed by a brief vortex. The mixture was pipetted up and down to homogenise the content. An amount of 800 µL of chloroform was added into the mixture (4 mL) and shaken vigorously, incubated for 10 min at room temperature, followed by a centrifuge at 12,000× *g* for 15 min at 4 °C. The organic and interphase layers were transferred into a 15 mL sterile falcon tube, and 1.2 mL of 100% ethanol (0.3 mL per 0.75 mL of Invitrogen™ TRIzol™ LS reagent) was added to the mixture. The tube was capped and was centrifuged several times until the DNA pellet was resuspended in 2 mL of 0.1 M sodium citrate in 10% ethanol, pH 8.5 (1 mL, per 0.75 mL of Invitrogen™ TRIzol™ LS Reagent) and transferred to a 2 mL eppendorf tube. The DNA pellet was resuspended in 2 mL of 75% ethanol (1.5–2 mL per 0.75 mL of Invitrogen™ TRIzol™ LS reagent used) and incubated for 20 min at room temperature. Finally, air-drying the DNA pellet for 5 min and washing the DNA was performed by resuspending the pellet in 20 µL Invitrogen™ TE Buffer (10 mM Tris-HCl (pH 8.0) and 0.1 mM EDTA).

DNA concentration and purity were checked on 1% agarose gels, and sterile water was used to dilute the DNA samples. DNA concentrations were determined using a Thermo Scientific™ NanoDrop™ 2000/2000c Spectrophotometer, Dover, DE, USA.

### 4.3. Library Preparation & Sequencing (V3–V4)

The variable regions, i.e., the V3–V4 area of the bacterial 16S ribosomal RNA (rRNA) gene, was amplified by polymerase chain reaction (PCR) using 515 forward (GGTGCCAGC MGCCGCGGTAA) and 806 reverse (GACTACHVG GGTWTCTAAT) primers Samples were prepared following the protocol in [48], using KAPA HiFi Polymerase to amplify variable region 3-4 of the 16S rRNA gene. Samples underwent 30 cycles of PCR. The libraries were sequenced at Diversigen (New Brighton, MN, USA) on an Illumina MiSeq using paired-end 2 × 250 reads with the MiSeq Reagent Kit V3 (Illumina, 600 cycle kit, San Diego, CA, USA). PCR controls included one negative and three positives (mock community, *E. coli* isolate and manufactured control for GC bias). All controls passed in the project.

### 4.4. Amplicon Sequence Variant (ASV) Picking (V4/V3V4)

Cutadapt was used to remove adaptors and primers from sequencing reads. The reads were quality checked by DADA2 (v1.25.2) R package [27214047], using default parameters of filterAndTrim function on forward and reverse reads; these specifically allowed for no ambiguous nucleotides (“maxN = 0”), truncated reads at the first instance of quality score < 2 (“truncQ = 2”) and allowed for maximum expected errors of less than 2 (“maxEE = c(2,2)”) where expected errors are calculated from the nominal definition of the quality score: EE = sum(10^^(−Q/10)^) by DADA2.

The error rates were calculated by the built-in machine learning function “learnErros” in DADA2. Then, forward and reverse reads were merged using the “mergePairs” function. Finally, chimeric reads were removed by “removeBimeraDenovo” function. As a result, two samples (samples no. 33 and 36) which had less than 1000 non-chimeric reads were removed. Next, by using the “assignTaxonomy” function of DADA2, the reads were mapped to the DADA2 formatted GTDB database (“GTDB_bac120_arc122_ssu_r202_fullTaxo.fa.gz”). The resulting taxa table, sequence table and associated meta-data were merged into the phyloseq object of 767 ASVs identified in a set of 29 individuals by phyloseq (1.42.0) package [23630581]. Eventually, 135 genera with minimum abundance greater than 0 were identified (Table S2—abundance counts) and 31 of them were observed >10% of the samples. Alpha diversity measures were calculated using the “diversity” function from the vegan R package [2.6–4]. Shannon and Simpson diversity per group were plotted using the “plot richness” function from Phyloseq. The diversity indices were compared by linear regression between benign and PDAC by adjusting for the number of non-chimeric reads for each sample. The principal components were extracted from central log-ratio-transformed (CLR) read counts (*n* + 1) using the “prcomp” function in R. PERMANOVA tests were calculated by adonis2 from vegan R package, while PERMADISP was calculated using betadisper (vegan), both adjusted by number nonchimeric reads per sample and 9999 using permutations. Linear regression analyses to test the association between PDAC and genera abundance were performed by Maaslin2 for common genera with abundance > 10 and by adjusting for non-chimeric reads per sample, as shown in Table S5. The Benjamini–Hochberg correction was used to correct for multiple testing. Patient characteristics were analysed on GraphPad Prism version 9.5.1. Independent *t*-test, Mann–Whitney U test and Chi-squared test were used to calculate the *p*-values. The full code scripts used in the analysis are made available in the Supplementary Materials.

## 5. Conclusions

Given the close relationship between microbiota and cancer, microbiome-targeted therapies are believed to be the next frontier in clinical cancer treatment. It also has a role in diagnostics and prognostication. Thus, with a greater understanding and definition of the bacterial microbiome in PDAC, there lies promise to develop novel biomarkers and therapeutic strategies. This study has demonstrated that patients with obstructive jaundice caused by PDAC have an altered microbiome in the bile compared to those with benign disease. We have identified four microbes that are associated with PDAC, and the genus *Streptococcus* (FDR = 0.033) was found to be of increased abundance in the PDAC group. Identification of specific bacteria in the bile may potentially enable the detection and stratification of PDAC. Patients undergoing biliary drainage could have bile analysed and “their microbial signature” targeted prior to surgery or neoadjuvant chemotherapy in order to optimise survival outcomes. Therefore, our study provides new insights into the link between the bile microbiome and PDAC. The results are promising and warrant a future larger study with metagenomic sequencing to investigate the function of these bacteria.

## Data Availability

All information is included in the manuscript or Supplementary Materials. The bioinformatic code is available in the supporting files. 16S V3/V4 sequencing data is deposited and available on NCBI BioProject Accession Number: PRJNA1018343.

## Supplementary Materials

The following supporting information can be downloaded at: https://www.mdpi.com/article/10.3390/ijms242316888/s1. The full scripts used in the analysis are available in the Supplementary Materials.
