# Supplementary material for: Bile Microbiome Signatures Associated with Pancreatic Ductal Adenocarcinoma Compared to Benign Disease: A UK Pilot Study
Source: Int J Mol Sci. 2023 Nov 28;24(23):16888. doi: 10.3390/ijms242316888 (PMC10706407; doi:10.3390/ijms242316888)
Supplement: Supplementary file 1 [file ijms-24-16888-s001.zip › Supplmentary Tables S1-S3.pdf]

Table S1. Summary of the patient cohort.

Variables	PDAC (n=12)	Benign cases (n= 17)	P-value
**Age, Years (median, range)**	71 (47-93)	64 (33-84)	0.330
**Sex, n, (%), Male: Female**	7 (59%): 5 (41%)	12 (70%): 5 (30%)	0.728
**DM, presence, n, (%)**	2 (17%)	4 (24%)	0.650
**Regular PPI use, n, (%)**	2 (17%)	4 (24%)	0.461
Never-smoked, **n, (%)**	6 (50%)	10 (59%)	
Ex-smoker, **n, (%)**	4 (33%)	4 (24%)	
Smoker, **n, (%)**	2 (17%)	3 (17%)	
**Bilirubin (μmol/L) (median, range)**	286 (115-428)	20.5 (9-94)	< 0.0001
**CRP (μmol/L) (median, range)**	23.15 (6.4-319.6)	10.3 (2 - 129.6)	0.3744
**CA-199 (median, range)**	1915 (201-84948)	9.5 (2 - 119)	<0.0001
**Preoperative biliary drainage, (n, %)**			
Yes	12 (100%)	4 (24%)	
No	0	13 (76%)	
**Type of procedure performed, n, (%)**			
ERCP	10 (83%)	17 (100%)	
PTBD	2 (17%)	0	
**Aetiology found at ERCP, n, (%)**			
No abnormality	n/a	2 (11%)	
Duct dilatation	n/a	2 (11%)	
Benign inflammatory stricture	n/a	4 (25%)	
Choledocholithiasis	n/a	7 (42%)	
Debris/grit/microlithiasis	n/a	2 (11%)	
Tumour location= Head	12 (100%)	n/a	
**Stage, n, (%)**			
IA	2 (17%)	n/a	
IB	1 (8%)	n/a	
IIA	3 (25%)	n/a	
IIB	0	n/a	
III	6 (50%)	n/a	
IV	0	n/a	

Table S2. We found four genera to be of significance among PDAC samples vs. benign adjusting for false discovery rate (FDR).

Variable	Beta	SE	P	FDR	FC	FC_lower	FC_upper
Bacteria_Proteobacteria_Enterobacterales_Enterobacteriaceae_Escherichia	-1.55672579	0.330932289	7.34E-05	0.002199348	0.339921663	0.216831599	0.532886983
Bacteria_Proteobacteria_Others_Others_Others1	-0.744065622	0.164563947	0.000118884	0.002199348	0.597054435	0.477439238	0.746637416
Bacteria_Proteobacteria_Enterobacterales_Enterobacteriaceae_Others	-1.791811134	0.480610494	0.000945937	0.011666553	0.288809252	0.150328692	0.554856049
Bacteria_Firmicutes_Lactobacillales_Streptococcaceae_Streptococcus	1.727734879	0.540590216	0.003638497	0.033656098	3.312073936	1.589064909	6.903326413

Table S3. MIMARKS Checklist.

Item	Description	MIMARKS survey	MIMARKS specimen
**Investigation**			
Submitted to INSDC	Sequences data has been submitted to SRA (Sequence Read Archives)	M	M
Investigation type	MIMARKS specimen		M
Project name	Bile Microbiome Signatures as Biomarkers for Differentiating Pancreatic Ductal Adenocarcinoma from Benign Disease: Discovery of novel microbial signatures in a UK pilot study.	M	M
**Environment**			
Geographic location (latitude and longitude [float, point, transect and region])	Geographical coordinates of Guildford, 51°14′11″N 0°34′13″W	M	M
Geographic location (depth [integer, point, interval, unit])	Geographical coordinates of Guildford, 51°14′11″N 0°34′13″W	E	E
Geographic location (elevation of site [integer, unit]; altitude of sample [integer, unit])	Geographical coordinates of Guildford, 51°14′11″N 0°34′13″W	E	E
Geographic location	United Kingdom	M	M
Collection date [ISO8601]	The time of sampling, 2021-2022	M	M
Environment (biome [EnvO])	biome	M	M
Environment (feature [EnvO])	n/a	M	M
Environment (material [EnvO])	n/a	M	M
**MIGS/MIMS/MIMARKS extension**			
Environmental package [air, host-associated, human-associated, human-skin,	Human-associated	M	M
**Nucleic acid sequence source**			
Isolation and growth conditions [PMID, DOI or URL]	Publication reference in the form of PubMed ID (PMID), digital object identifier (DOI) or URL for isolation and growth condition specifications of the organism/material	–	M
**Sequencing**			

Item	Description	MIMARKS survey	MIMARKS specimen
Target gene or locus (e.g., 16S rRNA, 18S rRNA, nif, amoA, rpo)	16S rRNA gene study	M	M
Sequencing method (e.g., dideoxysequencing, pyrosequencing, polony)	The variable regions V3-V4 area of the 16S ribosomal RNA (rRNA) gene was amplified by polymerase chain reaction (PCR) using bacterial and reverse primers. Samples were prepared with a protocol 22, using KAPA HiFi Polymerase to amplify variable region 4 (515f/806r) of the 16S rRNA gene. Samples underwent 30 cycles of PCR. Libraries were sequenced at Diversigen (New Brighton, MN, USA)	M	M
